# The impact of circadian rhythm on Bacillus Calmette-Guérin vaccination effects on SARS-CoV-2 infections

**DOI:** 10.3389/fimmu.2023.980711

**Published:** 2023-02-16

**Authors:** Konstantin Föhse, Esther J.M. Taks, Simone J. C. F. M. Moorlag, Marc J. M. Bonten, Reinout van Crevel, Jaap ten Oever, Cornelis H. van Werkhoven, Mihai G. Netea, Josephine S. van de Maat, Jacobien J. Hoogerwerf

**Affiliations:** ^1^ Department of Internal Medicine and Radboud Center for Infectious Diseases, Radboud University Medical Centre, Nijmegen, Netherlands; ^2^ Radboud Center for Infectious Diseases, Radboud University Medical Center, Nijmegen, Netherlands; ^3^ Julius Center for Health Sciences and Primary Care, University Medical Center Utrecht, Utrecht, Netherlands; ^4^ Department of Immunology and Metabolism, Life & Medical Sciences Institute, University of Bonn, Bonn, Germany

**Keywords:** COVID-19, respiratory tract infection, circadian rhythm, BCG, trained immunity, heterologous protection, SARS-CoV-2, circadian clock

## Abstract

**Background and objective:**

A recent study has suggested that circadian rhythm has an important impact on the immunological effects induced by Bacillus Calmette-Guérin (BCG) vaccination. The objective of this study was to evaluate whether the timing of BCG vaccination (morning or afternoon) affects its impact on severe acute respiratory syndrome–coronavirus-2 (SARS-CoV-2) infections and clinically relevant respiratory tract infections (RTIs).

**Methods:**

This is a *post-hoc* analysis of the BCG-CORONA-ELDERLY (NCT04417335) multicenter, placebo-controlled trial, in which participants aged 60 years and older were randomly assigned to vaccination with BCG or placebo, and followed for 12 months. The primary endpoint was the cumulative incidence of SARS-CoV-2 infection. To assess the impact of circadian rhythm on the BCG effects, participants were divided into four groups: vaccinated with either BCG or placebo in the morning (between 9:00h and 11:30h) or in the afternoon (between 14:30h and 18:00h).

**Results:**

The subdistribution hazard ratio of SARS-CoV-2 infection in the first six months after vaccination was 2.394 (95% confidence interval [CI], 0.856-6.696) for the morning BCG group and 0.284 (95% CI, 0.055-1.480) for the afternoon BCG group. When comparing those two groups, the interaction hazard ratio was 8.966 (95% CI, 1.366-58.836). In the period from six months until 12 months after vaccination cumulative incidences of SARS-CoV-2 infection were comparable, as well as cumulative incidences of clinically relevant RTI in both periods.

**Conclusion:**

Although there was a difference in effect between morning and afternoon BCG vaccination, the vaccine did not protect against SARS-COV-2 infections and clinically relevant RTI’s at either timepoint.

## Introduction

Trained immunity is an emerging concept which describes epigenetic, metabolic and functional reprogramming of innate immune cells that leads to an enhanced heterologous immune response to infections. Trained innate immune cells are characterized by an increased host defense function upon restimulation (higher cytokine production capacity, phagocytosis, intracellular pathogen killing). Bacillus Calmette-Guérin (BCG) is the vaccine against tuberculosis and has been shown to induce trained immunity and protect against heterologous infections (1). The magnitude of the heterologous responses induced by BCG is highly variable and dependent on many factors, including age, sex and environmental factors (2–6). A source of uncertainty is the duration of potential heterologous effects.

Recently, de Bree et al. have observed that the circadian rhythm may influence BCG-induced trained immunity (7). Three months after BCG vaccination, monocytes of individuals vaccinated between 8:00h and 9:00h produced higher cytokine levels upon ex-vivo stimulation compared to individuals vaccinated at 18:00h. These data support the large body of evidence suggesting that the innate immune system is under control of an intrinsic clock, similar to many functions in mammalian physiology. It has been hypothesized that endogenous oscillations of immune cells allow the host to anticipate variations and potential threats in the environment (8). For example, synchronizing the magnitude of the immune response against foodborne pathogens with the phase of feeding is much more energy efficient than synchronizing it with the phase of sleeping (9).

The protective effects of trained immunity and BCG have been shown against a variety of viral pathogens (10–13). Therefore, before specific SARS-CoV-2 vaccines were developed and became available, it was suggested BCG might provide some protection against Coronavirus disease 2019 (COVID-19). However, clinical trials on the effect of BCG on respiratory tract infections (RTIs) including COVID-19 produced mixed results. Two trials on BCG re-vaccination performed in Greek elderly populations resulted in lower numbers of RTIs of probable viral origin and COVID-19, respectively (14, 15). On the other hand, trials on BCG vaccination performed in Dutch elderly cohorts and in Dutch healthcare workers did not result in lower numbers of RTIs or COVID-19 (16, 17). Given the earlier report of a circadian rhythm effect on BCG immunological effects, with the strongest effect in the morning, we analyzed whether the time of the day of vaccination in one of these clinical trials (the BCG-CORONA-ELDERLY study) influenced the effect on susceptibility to infections. While the overall analysis in this trial found no significant effect of BCG vaccination on the incidence of RTI or COVID-19, one could envisage that an effect may be observed in the sub-group of volunteers vaccinated in the morning.

## Methods

The present analysis is a sub-study of the BCG-CORONA-ELDERLY study (NCT04417335), a prospective, randomized, placebo-controlled trial in two tertiary centers in the Netherlands. For an extensive description of the protocol, methods and results, please refer to the protocol (18) and the clinical data reported by Moorlag et al. (16).

In short, 2014 immunocompetent individuals with a median age of 67 years (interquartile range 64–72 years) were included in the original trial. Participants were randomly assigned in a 1:1 ratio to receive either 0.1 mL of BCG (Danish strain 1331, SSI, Denmark) or 0.1 mL placebo (0.9% NaCl solution) *via* intradermal injection.The time slot of vaccination was randomly assigned. If participants were unable to attend at that time, they were allowed to reschedule their appointment. Subsequently, participants had to document clinical symptoms, COVID-19 testing, COVID-19 exposure, and visits to healthcare professionals in the 12 months following vaccination.

To analyze the effect of the circadian rhythm on the clinical outcomes, all participants were divided into four groups based on the time of vaccination (morning or afternoon) and based on the intervention (BCG or placebo). Participants in the morning were vaccinated between 9:00h and 11:30h and participants in the afternoon were vaccinated between 14:30h and 18:00h. Individuals vaccinated between 11:30h and 14:30h were not analyzed in the current study. We chose for those time intervals to obtain two groups of comparable size at the two ends of the vaccination time of the day. Furthermore, those intervals give a realistic reflection of common practice of vaccination. The time of randomization was regarded as the time of vaccination, since randomization was precisely registered in the database and randomization was usually not more than 5 minutes apart from the vaccination. Next to the full 12 months follow-up, we separately analyzed the periods from vaccination until six months and from six months until 12 months after vaccination to account for trained immunity effects that are much stronger in the initial months after vaccination. The primary outcome of our study was the cumulative incidence of SARS-CoV-2 infection, detected by a polymerase chain reaction (PCR) test and irrespective of symptoms. Secondary endpoint was the cumulative incidence of clinically relevant respiratory tract infection. In order to meet this criterion, participants had to have at least one respiratory symptom and one systemic symptom that required medical intervention within 5 days of the onset of symptoms. Medical interventions included the start or change in antibiotic, antiviral, corticosteroid or pulmonary treatment, or hospital admission.

The endpoints were analyzed using the Fine and Gray competing risks proportional hazards model. Time to event was defined as the dependent outcome, time of vaccination as the independent variable, and mortality as potential competing event. The model was adjusted for the participating hospital and statistically different baseline characteristics that were tested by analysis of variance and included age category, BMI and hypertension. The effect for both endpoints was reported as a hazard ratio with 95% confidence interval (CI). Participants who met one of the endpoints in the first six months after vaccination were removed from the analysis of the second part of the year.

First, we compared the group vaccinated with BCG in the morning with the group vaccinated with placebo in the morning, and the group vaccinated with BCG in the afternoon with the group vaccinated with placebo in the afternoon. Second, we compared the two BCG groups with each other. Data were analyzed using R version 4.1.1 (19).

## Results

Patient characteristics are shown in **Table 1**. Among the volunteers participating in the study, 358 participants were vaccinated with BCG and 356 with placebo between 9:00h and 11:30h, and 320 participants were vaccinated with BCG and 309 with placebo between 14:30h and 18:00h. The median age of all groups was 67 years, although adults over the age of 80 were more prevalent in the morning groups compared to the afternoon groups (p<0.01). In the morning placebo group were more males than females, whereas in the afternoon placebo group more females were present. In the BCG groups the sex distribution was balanced. Participants vaccinated with BCG in the afternoon had a slightly higher BMI than the participants of the other groups (p=0.04). Hypertension was less prevalent amongst participants who got vaccinated with BCG in the morning compared to those who got vaccinated with placebo in the morning (p=0.03), and those who got vaccinated with BCG in the afternoon (p=0.01). The remaining baseline characteristics were comparable in all groups (**Table 1**).

**Table 1:** Baseline characteristics of study participants.

Variable	BCG in the morning (N=358)	Placebo in the morning (N=356)	BCG in the afternoon (N=320)	Placebo in the afternoon (N=309)	p-value BCG morning – BCG afternoon	p-value BCG morning – placebo morning	p-value BCG afternoon – placebo afternoon
Median age (IQR) – yr	67 (64-72)	67 (64-72)	67 (64-70)	67 (63-70)	0.047	0.545	0.720
Age category – no. (%)							
60 – 69 yr	221 (61.7)	230 (64.6)	225 (70.3)	211 (68.3)	0.019	0.426	0.581
70 – 79	109 (30.4)	99 (27.8)	86 (26.9)	90 (29.1)	0.305	0.437	0.530
80+	28 (7.8)	27 (7.6)	9 (2.8)	8 (2.6)	0.004	0.906	0.863
Sex – no. (%)							
Male sex	176 (49.2)	193 (54.2)	163 (50.9)	139 (45.0)	0.644	0.177	0.135
Female sex	182 (50.8)	163 (45.8)	157 (49.1)	170 (55.0)			
Median BMI (IQR) – kg/m^2^	24.8 (22.9-27.4)	24.8 (23.1-27.4)	25.7 (23.5-28.1)	25.1 (23.3-28.1)	0.015	0.827	0.537
Comorbidities							
Cardiovascular disease	65 (18.2)	73 (20.5)	51 (15.9)	52 (16.8)	0.444	0.427	0.763
Hypertension	89 (24.9)	115 (32.3)	110 (34.4)	89 (29.0)	0.007	0.028	0.133
Diabetes	29 (8.1)	20 (5.6)	21 (6.6)	20 (6.5)	0.444	0.190	0.964
Asthma	17 (4.7)	19 (5.3)	17 (5.3)	20 (6.5)	0.737	0.719	0.537
Other pulmonary disease	9 (2.5)	12 (3.4)	9 (2.8)	8 (2.6)	0.809	0.498	0.863
Renal disease	3 (0.8)	9 (2.5)	8 (2.5)	8 (2.6)	0.087	0.079	0.944
Allergic rhinitis	81 (22.6)	92 (25.8)	70 (21.9)	75 (24.3)	0.815	0.316	0.476
Use of any medication – no. (%)	246 (68.7)	250 (70.2)	220 (68.8)	219 (70.9)	0.992	0.661	0.562
Median number of daily used medication (IQR)	1.0 (0.0-3.0)	2.0 (0.0-3.0)	1.0 (0.0-3.0)	2.0 (0.0-4.0)	0.524	0.154	0.488
Never smoked	124 (34.6)	137 (38.5)	102 (31.9)	108 (35.0)	0.446	0.286	0.413
Past smoking	219 (61.2)	199 (55.9)	199 (62.2)	185 (59.9)	0.786	0.153	0.551
Current smoking	13 (3.6)	18 (5.1)	19 (5.9)	15 (4.9)	0.158	0.350	0.548
Second hand smoke	2 (0.6)	1 (0.3)	0	1 (0.3)	0.181	0.566	0.309
BCG vaccination history – no. (%)							
Unknown	49 (13.7)	64 (18.0)	64 (20.0)	67 (21.7)	0.028	0.116	0.732
No	204 (57.0)	196 (55.1)	160 (50.0)	156 (50.5)	0.069	0.604	0.903
Yes	105 (29.3)	94 (26.4)	95 (29.7)	86 (27.8)	0.919	0.383	0.607
Median years since BCG vaccination (IQR)	50 (45-56)	48 (42.3-55)	49 (44-53)	49 (44.5-58)	0.325	0.146	0.365
SARS-CoV-2 vaccination history – no. (%)							
Vaccinated	358 (100)	355 (99.7)	318 (99.4)	307 (99.4)	0.134	0.156	0.972
Pfizer/BioNTech	252 (70.4)	248 (69.7)	217 (67.8)	196 (63.4)	0.468	0.832	0.247
AstraZeneca	99 (27.7)	100 (28.1)	95 (29.7)	106 (34.3)	0.559	0.897	0.215
Moderna	6 (1.7)	6 (1.7)	3 (0.9)	4 (1.3)	0.402	0.992	0.670
Janssen	1 (0.3)	1 (0.3)	2 (0.6)	1 (0.3)	0.498	0.997	0.583
Median days until first SARS-CoV-2 vaccination (IQR)	346 (332.8-358)	347 (333.8-358)	349 (332-358)	350 (335-359)	0.288	0.286	0.292
Other vaccines – no. (%)							
Live vaccines in the past year	3 (0.8)	2 (0.6)	1 (0.3)	2 (0.6)	0.372	0.658	0.524
Non-live vaccines in the past year	251 (70.1)	242 (68.0)	226 (70.6)	221 (71.5)	0.884	0.537	0.804

BCG, Bacillus Calmette-Guérin; CI, confidence interval; BMI, body mass index.

In the first six months after vaccination, the cumulative incidence of SARS-CoV-2 infection was 0.014 (95% CI 0.005-0.031) in the placebo morning group and 0.034 (95% CI 0.018-0.056) in the BCG morning group (subdistribution hazard ratio [SDHR] 2.394, 95% CI 0.856-6.696) (**Table 2A**). In the afternoon results are in the opposite direction, but not statistically significant (SDHR 0.284, 95% CI 0.055-1.480). When comparing the BCG morning and afternoon group with each other, the interaction hazard ratio [IHR] is 8.966 (95% CI 1.366-58.836), indicating a difference in effect between the two timepoints. In the second part of the year, cumulative incidences were more comparable with SDHRs of 0.745 (95% CI 0.437-1.600) and 1.460 (95% CI 0.505-4.223) for the morning and the afternoon group, respectively (**Table 2B**). The IHR of the two BCG groups is 0.530 (95% CI 0.149-1.881). The analysis of the full 12 months follow-up is in line with the aforementioned and did not reveal any statistically significant differences in the cumulative incidence of SARS-CoV-2 infection (**Table 2C**).

Table 2AEffect of morning and afternoon BCG vaccination compared to placebo in the first six months after vaccination.

**Table:** 

Subgroup	Intervention	Follow-up time (years)	Events (n)	Cumulative incidence	SDHR	IHR
SARS-CoV-2 infections						
Morning	Placebo	168.5	5	0.014 (0.005-0.031)	[ref]	–
	BCG	171.3	12	0.034 (0.018-0.056)	2.394 (0.856-6.696)	8.966 (1.366-58.836)
Afternoon	Placebo	149.7	7	0.023 (0.010-0.045)	[ref]	–
	BCG	153.4	2	0.006 (0.001-0.021)	0.284 (0.055-1.480)	[ref]
Clinically relevant RTIs						
Morning	Placebo	173.9	3	0.009 (0.002-0.023)	[ref]	–
	BCG	174.6	4	0.011 (0.004-0.027)	1.510 (0.367-6.207)	0.351 (0.025-4.978)
Afternoon	Placebo	150.4	1	0.003 (0.000-0.017)	[ref]	–
	BCG	155.2	4	0.013 (0.004-0.030)	4.916 (0.569-42.461)	[ref]

BCG, Bacillus Calmette-Guérin; RTI, Respiratory tract infection; SARS-CoV-2, Severe acute respiratory syndrome coronavirus 2; CI, confidence interval; SDHR, subdistribution hazard ratio; IHR, interaction hazard ratio.

Cumulative incidences and hazard ratios are reported with 95% confidence interval.

Table 2BEffect of morning and afternoon BCG vaccination compared to placebo in the period from six months to 12 months after vaccination.

**Table:** 

Subgroup	Intervention	Follow-up time (years)	Events (n)	Cumulative incidence	SDHR	IHR
Clinically relevant RTIs						
Morning	Placebo	171.0	4	0.011 (0.004-0.028)	[ref]	–
	BCG	172.4	7	0.020 (0.009-0.039)	1.748 (0.524-5.828)	2.260 (0.376-13.571)
Afternoon	Placebo	144.7	5	0.017 (0.006-0.037)	[ref]	–
	BCG	150.8	4	0.013 (0.004-0.031)	0.805 (0.199-3.249)	[ref]
SARS-CoV-2 infections						
Morning	Placebo	165.8	16	0.046 (0.027-0.072)	[ref]	–
	BCG	167.1	12	0.035 (0.019-0.058)	0.745 (0.437-1.600)	0.530 (0.149-1.881)
Afternoon	Placebo	141.9	6	0.021 (0.009-0.042)	[ref]	–
	BCG	149.5	9	0.029 (0.014-0.052)	1.460 (0.505-4.223)	[ref]

BCG, Bacillus Calmette-Guérin; RTI, Respiratory tract infection; SARS-CoV-2, Severe acute respiratory syndrome coronavirus 2; CI, confidence interval; SDHR, subdistribution hazard ratio; IHR, interaction hazard ratio.

Cumulative incidences and hazard ratios are reported with 95% confidence interval.

Table 2CEffect of morning and afternoon BCG vaccination compared to placebo in the full 12 months follow-up.

**Table:** 

Subgroup	Intervention	Follow-up time (years)	Events (n)	Cumulative incidence	SDHR	IHR
Clinically relevant RTIs						
Morning	Placebo	348.6	7	0.020 (0.009-0.039)	[ref]	–
	BCG	351.2	11	0.031 (0.016-0.053)	1.616 (0.646-4.046)	1.218 (0.295-5.037)
Afternoon	Placebo	299.4	6	0.020 (0.008-0.041)	[ref]	–
	BCG	310.4	8	0.026 (0.012-0.048)	1.417 (0.468-4.296)	[ref]
SARS-CoV-2 infections						
Morning	Placebo	344.0	21	0.060 (0.038-0.088)	[ref]	–
	BCG	309.8	24	0.067 (0.044-0.097)	1.160 (0.641-2.098)	1.422 (0.527-3.832)
Afternoon	Placebo	296.4	13	0.043 (0.024-0.071)	[ref]	–
	BCG	309.8	11	0.035 (0.019-0.060)	0.837 (0.368-1.902)	[ref]

BCG, Bacillus Calmette-Guérin; RTI, Respiratory tract infection; SARS-CoV-2, Severe acute respiratory syndrome coronavirus 2; CI, confidence interval; SDHR, subdistribution hazard ratio; IHR, interaction hazard ratio.

Cumulative incidences and hazard ratios are reported with 95% confidence interval.

Due to the interventions of the COVID-19 pandemic, such as quarantine, isolation, and social distancing, the number of clinically relevant RTIs was much lower than SARS-CoV-2 infections. The SDHR was comparable in all time periods (**Table 2A–C**).

In conclusion, neither participants vaccinated with BCG in the morning nor in the afternoon were protected against respiratory infections including SARS-CoV-2.

## Discussion

The results of the present study show that the time of day of BCG vaccination did not affect the susceptibility to respiratory infections. We observed some differences in the cumulative incidence of SARS-CoV-2 infections, especially in the first six months after vaccination, but the number of events was too low and consequently confidence intervals were too wide to draw any conclusion. Notably, the direction of the effects was even in the opposite direction of our initial hypothesis that BCG vaccination offers better protection in the morning. It is important mentioning that the initial trial was not powered nor designed to analyze the effect of circadian rhythm. The most likely explanation for our findings is that BCG vaccination simply has no effect on the protection against RTIs and SARS-CoV-2 infections in this study. A protective effect has previously been demonstrated in several smaller studies (14, 20–22), but pathophysiological differences between SARS-CoV-2 infections and other RTIs (such as influenza) may account for these differential effects of BCG (23). Another explanation why our results contradict those from de Bree et al. may be that in their experiments the time period between vaccination and blood collection was just three months, and that the morning group was vaccinated between 8:00 and 9:00 and the afternoon group at 18:00 (7).

We have chosen for greater intervals of vaccination in this study to give a better reflection of common practice of vaccination and to guarantee a certain number of events per group. Furthermore, the median age of the individuals in the study from de Bree et al. was 26 years, while in contrast, the median age in our cohort was 67. Ageing has been associated with both changes in circadian-influenced biological processes and innate immune responses (20). Lastly, de Bree et al. observed the trained immunity effects upon stimulation with bacterial stimuli, whereas SARS-CoV-2 and the most common RTIs are viral infections. One could also speculate whether the difference in cytokine response between the morning and the afternoon is too small to affect clinical outcomes. *In-vitro* effects often do not correspond with clinical significance and generally need to be interpreted with care (21).

A protective effect has previously been demonstrated in several (smaller) studies (14, 20–22), but pathophysiological differences between SARS-CoV-2 infections and other RTIs (such as influenza) may account for these differential effects of BCG (23). Our study is the first to evaluate the clinical impact of circadian rhythm on BCG effects in humans. Research on the impact of the circadian clock on other vaccines is also limited to experimental data and focuses on adaptive immune responses. Clinical trials are generally lacking. However, studies on influenza, hepatitis A and SARS-CoV-2 found either no effect of circadian rhythm on antibody titers or mostly better efficacy when administrating vaccines in the morning (22–24).

On the cellular level, virtually all cell lines of the innate and the adaptive immune system have been associated with circadian variations (25). This is also the case for myeloid cells, natural killer cells and innate lymphoid cells, all of which are involved in the induction of trained immunity. The same effects have been demonstrated for metabolic processes and the expression of pathogen recognition receptors, such as TLR-9 (26, 27). Both mechanisms also play a role in the induction of innate immune responses and have been identified to be under control of a molecular clock. It has been suggested that fluctuating cytokine and cortisol levels may be the underlying mechanism for the effect of circadian rhythm on vaccine immunogenicity, as they are both known to be potent regulators of immune functions (28, 29). Taken together, these formed the basis of our hypothesis that the circadian clock also affects trained immunity (7).

Although we did not detect that BCG vaccination, either in the morning or the afternoon, offers beter protection than placebo against RTIs or COVID-19, we believe that further unravelling the mechanisms of clock-controlled immunomodulation has the potential to enhance both the immunogenicity of vaccines and the efficacy of immunotherapies. Therefore, further research is needed to identify potential clinical implications of the previously found *in vitro* effects on immune response.

## Data availability statement

The raw data supporting the conclusions of this article will be made available by the authors, without undue reservation.

## Ethics statement

The studies involving human participants were reviewed and approved by Arnhem-Nijmegen Ethical Committee (# NL73430.091.20). The patients/participants provided their written informed consent to participate in this study.

## Author contributions

KF and ET wrote the manuscript. KF, ET, and CvW analyzed the data and performed the statistical analysis. ET and SM conducted the study. MB, RvC, JtO, CvW, MN, JvdM, and JH conceptualized the study and participated in writing the manuscript. All authors contributed to the article and approved the submitted version.
