# Corrigendum: The impact of circadian rhythm on Bacillus Calmette-Guérin vaccination effects on SARS-CoV-2 infections

**DOI:** 10.3389/fimmu.2023.1208659

**Published:** 2023-05-24

**Authors:** Konstantin Föhse, Esther J. M. Taks, Simone J. C. F. M. Moorlag, Marc J. M. Bonten, Reinout van Crevel, Jaap ten Oever, Cornelis H. van Werkhoven, Mihai G. Netea, Josephine S. van de Maat, Jacobien J. Hoogerwerf

**Affiliations:** ^1^ Department of Internal Medicine and Radboud Center for Infectious Diseases, Radboud University Medical Centre, Nijmegen, Netherlands; ^2^ Radboud Center for Infectious Diseases, Radboud University Medical Center, Nijmegen, Netherlands; ^3^ Julius Center for Health Sciences and Primary Care, University Medical Center Utrecht, Utrecht, Netherlands; ^4^ Department of Immunology and Metabolism, Life & Medical Sciences Institute, University of Bonn, Bonn, Germany

**Keywords:** COVID-19, respiratory tract infection, circadian rhythm, BCG, trained immunity, heterologous protection, SARS-CoV-2, circadian clock

In the published article, there was an error. We stated that in the first six month after vaccination, BCG vaccination in the afternoon offered better protection against SARS-CoV-2 infections than BCG vaccination in the morning. Given the lack of statistical significance in the analysis comparing the BCG-vaccinated individuals to those who received placebo in the morning and afternoon, it is not accurate to use ‘protection’ when describing the significant interaction hazard ratio in **Table 2A**.

A correction has been made to the conclusion of the Abstract. This sentence previously stated:

“Vaccination with BCG in the afternoon offered better protection against SARS-CoV-2 infections than BCG vaccination in the morning in the first six months after vaccination.”

The corrected sentence appears below:

“Although there was a difference in effect between morning and afternoon BCG vaccination, the vaccine did not protect against SARS-COV-2 infections and clinically relevant RTI’s at either timepoint.”

A correction has been made to the last three paragraphs of the results. The paragraphs previously stated:

“In the first six months after vaccination, the cumulative incidence of SARS-CoV-2 infection was significantly lower in the BCG afternoon group compared to the BCG morning group (interaction hazard ratio [IHR] 8.966, 95% CI 1.366-58.836) (**Table 2A**). The cumulative incidence of SARS-CoV-2 infection in the BCG afternoon group also tended to be lower than in the respective placebo group, although not statistically significant (subdistribution hazard ratio [SDHR] 0.284, 95% CI 0.055-1.480). In the morning, results are in the opposite direction, and the placebo group tended to be better protected against SARS-CoV-2 (SDHR 2.394, 95% CI 0.856-6.696). In the period from six months after vaccination until 12 months after vaccination cumulative incidences were comparable (**Table 2B**). The SDHR of SARS-CoV-2 infections was 1.460 (95% CI 0.505-4.223) for the afternoon BCG group and 0.745 (95% CI 0.43-1.600) for the morning BCG group (IHR 0.530, 95% CI 0.149-1.881). A better protection of BCG vaccination in the afternoon against SARS-CoV-2 is also reflected in the analysis of the full 12 months follow-up (cumulative incidences 0.035 [95% CI 0.019-0.060] in the afternoon versus 0.067 [95% CI 0.044-0.097] in the morning), but neither statistically significant (**Table 2C**).

Due to the interventions of the COVID-19 pandemic, such as quarantine, isolation, and social distancing, the number of clinically relevant RTIs was much lower than SARS-CoV-2 infections. The IHR of the morning BCG group was 0.351 (95% CI 0.025-4.978) in the first part of the year, 2.260 (95% CI 0.376-13.571) in the second part of the year and 1.218 (95% CI 0.295-5.037) in the analysis of the full 12 months (**Tables 2A-C**).

In conclusion, BCG vaccination in the afternoon in the first six months offered better protection than BCG vaccination in the morning against SARS-CoV-2 infections, and potentially better protection than placebo vaccination in the afternoon.”

The corrected paragraphs appears below:

“In the first six months after vaccination, the cumulative incidence of SARS-CoV-2 infection was 0.014 (95% CI 0.005-0.031) in the placebo morning group and 0.034 (95% CI 0.018-0.056) in the BCG morning group (subdistribution hazard ratio [SDHR] 2.394, 95% CI 0.856-6.696) (**Table 2A**). In the afternoon results are in the opposite direction, but not statistically significant (SDHR 0.284, 95% CI 0.055-1.480). When comparing the BCG morning and afternoon group with each other, the interaction hazard ratio [IHR] is 8.966 (95% CI 1.366-58.836), indicating a difference in effect between the two timepoints. In the second part of the year, cumulative incidences were more comparable with SDHRs of 0.745 (95% CI 0.437-1.600) and 1.460 (95% CI 0.505-4.223) for the morning and the afternoon group, respectively (**Table 2B**). The IHR of the two BCG groups is 0.530 (95% CI 0.149-1.881). The analysis of the full 12 months follow-up is in line with the aforementioned and did not reveal any statistically significant differences in the cumulative incidence of SARS-CoV-2 infection (**Table 2C**).

Due to the interventions of the COVID-19 pandemic, such as quarantine, isolation, and social distancing, the number of clinically relevant RTIs was much lower than SARS-CoV-2 infections. The SDHR was comparable in all time periods (**Tables 2A–C**).

In conclusion, neither participants vaccinated with BCG in the morning nor in the afternoon were protected against respiratory infections including SARS-CoV-2.”

A correction has been made to the first two paragraphs of the Discussion. The paragraphs previously stated:

“The results of the present study show that, in the first six months after vaccination, BCG vaccination in the afternoon offered better protection against SARS-CoV-2 infections than BCG vaccination in the morning. In addition, BCG vaccination in the afternoon tended to offer better protection against SARS-CoV-2 than placebo. We did not observe those effects in the period from six months after vaccination until one year after vaccination. Our results should be interpreted with caution as this trial was not powered nor designed to analyze the effect of circadian rhythm. Consequently, the number of events per subgroup was low and confidence intervals were wide. Despite this limitation, the results argue against our initial hypothesis of a stronger heterologous effect of BCG in the morning. The time of BCG vaccination did not affect the impact on clinically relevant RTIs.

Our finding of a potential better protection of BCG in the afternoon contradicts the experimental results from de Bree et al (7). Possible explanations may be that in the experimental study from de Bree et al. the time period between vaccination and blood collection was just three months, and that the morning group was vaccinated between 8:00 and 9:00 and the afternoon group at 18:00. …”

The corrected paragraphs appears below:

“The results of the present study show that the time of day of BCG vaccination did not affect the susceptibility to respiratory infections. We observed some differences in the cumulative incidence of SARS-CoV-2 infections, especially in the first six months after vaccination, but the number of events was too low and consequently confidence intervals were too wide to draw any conclusion. Notably, the direction of the effects was even in the opposite direction of our initial hypothesis that BCG vaccination offers better protection in the morning. It is important mentioning that the initial trial was not powered nor designed to analyze the effect of circadian rhythm.

The most likely explanation for our findings is that BCG vaccination simply has no effect on the protection against RTIs and SARS-CoV-2 infections in this study. A protective effect has previously been demonstrated in several smaller studies (14, 20-22), but pathophysiological differences between SARS-CoV-2 infections and other RTIs (such as influenza) may account for these differential effects of BCG (23). Another explanation why our results contradict those from de Bree et al. may be that in their experiments the time period between vaccination and blood collection was just three months, and that the morning group was vaccinated between 8:00 and 9:00 and the afternoon group at 18:00 (7).”

A correction has been made to the third paragraph of the Discussion. The following sentences were removed:

“A trend towards lower incidence of SARS-CoV-2 infections in the BCG afternoon group compared to the placebo group in the first six months, may also point to a – if at all – better protection of BCG vaccination in the afternoon. An explanation for the absence of a clear effect may be that BCG vaccination has no effect on the protection against SARS-CoV-2 infections which makes the timing of the vaccination irrelevant.”

A correction has been made to the third paragraph of the Discussion. The following sentence was moved further up:

“A protective effect has previously been demonstrated in several (smaller) studies (14, 20-22), but pathophysiological differences between SARS-CoV-2 infections and other RTIs (such as influenza) may account for these differential effects of BCG (23).”

A correction has been made to the fourth paragraph of the Discussion, paragraph 4. The following sentence was removed:

“Interestingly, we only observed a trend towards protective effects of BCG only in the first six months after vaccination, which could mean that trained immunity effects are relatively short-lasting.”

The authors apologize for these errors and state that this does not change the scientific conclusions of the article in any way. The original article has been updated.

